# 

**Published:** 1890-06-21

**Authors:** 


PRICE ONE PENNY.

No. 195, Vol. VIII.—June 21st, 1890.

[Registered at the General Post Office as a Newspaper for transmission in the United Kingdom and Abroad.]

WITH EXTRA NURSING SUPPLEMENT.
Per Annum, 6s. 6d.; post free.
Monthly Parts, 6d.; post free 8d.
Ditto per Annum, 8s., post free.

